# Transcultural Adaptation and Validation of the Spanish Version of the Sexual Satisfaction Scale for Women (SSS-W-E)

**DOI:** 10.3390/ijerph18189663

**Published:** 2021-09-14

**Authors:** Regina Ruiz de Viñaspre-Hernández, Rosana Garrido-Santamaria, Raquel Urra-Martínez, Paula Sáenz-Cabredo, Jesús Martínez-Tofe, Amaya Burgos-Esteban, Vicente Gea-Caballero, Isabel Antón-Solanas, Iván Santolalla-Arnedo, Raúl Juárez-Vela

**Affiliations:** 1 Department of Nursing, University of La Rioja, 26004 Logroño, La Rioja, Spain; reruizde@unirioja.es (R.R.d.V.-H.); amaya.burgos@unirioja.es (A.B.-E.); raul.juarez@unirioja.es (R.J.-V.); 2 Biomedical Research Center of La Rioja (CIBIR), Healthcare System Sustainability Research Unit (GISOSS), 26004 Logroño, La Rioja, Spain; tofe79@hotmail.com; 3 Government of La Rioja, Planificación Center, 26004 Logroño, La Rioja, Spain; rgarridos@riojasalud.es (R.G.-S.); rurra@riojasalud.es (R.U.-M.); pmsaenz@riojasalud.es (P.S.-C.); 4 Government of La Rioja, Hospital San Pedro, 26004 Logroño, La Rioja, Spain; 5 Faculty of Health Sciences, International University of Valencia, 46002 Valencia, Spain; 6 Department of Physiatry and Nursing, Faculty of Health Sciences, University of Zaragoza, 50009 Zaragoza, Spain; ianton@unizar.es

**Keywords:** sexual satisfaction, women’s sexual health, female sexual dysfunction, sexual behavior

## Abstract

Background: Sexual satisfaction is a complex and multidimensional concept. It encompasses physical, emotional, relational and cultural dimensions, and constitutes an essential component of sexual health, as well as an indicator of quality of life and wellbeing. The Sexual Satisfaction Scale for Women (SSS-W) was designed in the United States, and it is a valid and reliable tool to measure women’s sexual satisfaction. Aim: The aim of this study was to culturally adapt and translate the SSS-W into Spanish and analyze its psychometric properties. Methods: First, the original instrument was culturally adapted and translated from English to Spanish. Then, we tested the psychometric properties of the instrument in its Spanish version in a sample of 316 women who attended a family planning clinic in Logroño, Spain. Internal consistency reliability of the whole scale and each subscale separately was measured using Cronbach’s alpha. Factorial validity of the SSS-W in its Spanish version was analyzed using exploratory factor analysis through the Kaiser–Meyer–Olkin measure of sample adequacy and Bartlett’s Sphericity test. Results: The Cronbach’s alpha coefficients of the total scale and each subscale were satisfactory (>0.7). Exploratory factor analysis confirmed the five hypothetical dimensions of the scale in its Spanish version. The five dimensions (contentment, communication, compatibility, relational concern, and personal concern) explained 60% of the total variance of the scale; factor analysis using varimax rotation revealed strong loads in each of the five components. Conclusions: The SSS-W in its Spanish version is a valid and reliable tool to assess sexual satisfaction in Spanish women of reproductive age and, therefore, can be used both in clinical practice and for the investigation of sexual health.

## 1. Introduction

Sexual satisfaction is a complex and multidimensional concept; it is a subjective evaluation of a person’s likes and dislikes about their sexual life, as well as an effective response that arises from the evaluation of the positive and negative aspects associated with sexual activity or, in other words, the ability of an individual to derive pleasure from sexual activity [1,2]. It encompasses physical, emotional, relational and cultural dimensions and is an essential component of sexual health and an indicator of people’s quality of life and wellbeing [3]. According to previous studies, women’s sexual satisfaction is influenced by a range of factors, namely age, marital status, educational level, income [4,5], physical and psychological health status [6], personality [7], beliefs, cultural values and attitudes associated with sexuality [8], sexual behavior [9], characteristics of the affective relationship with the partner—level of satisfaction and commitment [10,11]—and the existence of sexual difficulties [12]. Sexual satisfaction is an important indicator of sexual health and is strongly associated with women’s satisfaction with their affective relationships, even in cultures as different as Spain [11] and China [13]. In women, the perception of sexual satisfaction has both personal and relational components. Personal components are associated with individual and positive experiences of sexuality, where pleasure and pleasant feelings provide personal sexual wellbeing. Relational components are manifested in experiences of reciprocity, communication, romance, expression of feelings, creativity, a manifestation of desires, and frequency of sexual activity [14]. The degree of sexual satisfaction that women experience is decisive in their perception of the quality of their affective-sexual relationships [15].

The Sexual Satisfaction Scale for Women (SSS-W) is a comprehensive, valid, and reliable self-report measure of women’s sexual satisfaction. The initial version of the scale comprising 22 items was derived from a literature review and tested on a sample of 538 women. Three domains were identified after exploratory factor analysis: two relational (communication and sexual compatibility) and one personal (sexual satisfaction). Subsequently, the domains concern about the relationship and personal concern were added based on the information obtained through interviews with women with diagnosed sexual dysfunction. The final version included 30 items classified into five domains of six items each: contentment, communication, compatibility, concern about the relationship, and personal concerns. This version was validated in a sample of North American women and showed good ability to discriminate between women with and without sexual dysfunction [16]. The scale has subsequently been translated and adapted for use in other languages, including Traditional Chinese spoken in Taiwan [17,18] and Portuguese spoken in Brazil [19], and has been used to evaluate sexual satisfaction in previous studies [10,20,21,22].

The perception, experience and expression of sexual satisfaction in women are highly influenced by language and culture [23]. More than 585 million people, 7.5% of the world’s population, spoke Spanish at the beginning of 2020 and, after English, Spanish is the second most frequently used language in scientific communication [24]. However, to our knowledge, the Spanish version of the SSS-W has not yet been developed. Therefore, the purpose of this study was to translate, culturally adapt, and evaluate the psychometric properties, reliability and construct validity, of the Spanish version of the SSS-W.

## 2. Materials and Methods

### 2.1. Translation and Cultural Adaptation

First, the original SSS-W was translated and culturally adapted from English into Spanish. Permission to translate and culturally adapt the original tool into Spanish was sought and obtained from the authors [16]. We used the six-step procedure proposed by Beaton et al. [25]: (1) initial translation, (2) synthesis, (3) back translation, (4) back translation synthesis, (5) expert committee review of the translated version and (6) preliminary tests. Accordingly, the original SSS-W was translated into Spanish by two independent translators: an expert in medical translation and a researcher who was familiar with the instrument and its characteristics. The translators were instructed to use simple sentences and avoid metaphors, colloquial terminology, passive sentences, and hypothetical statements. Subsequently, both forward translations of the scale were assessed by an experts committee comprising the authors of this manuscript and two lecturers in women’s health who were proficient users of English and Spanish and who had previous clinical experience in the field of women’s health. During this session, the differences between the two translated versions were discussed and the first Spanish version of the SSS-W was obtained. This first Spanish version of the tool was back translated into English by a researcher who was a native speaker of both English and Spanish and who had not seen the original version of the SSS-W. Minor translation problems were solved by email, obtaining the new English version of the instrument. This new English version of the SSS-W was submitted to the author of the original instrument, who confirmed the accuracy of the back translation. The expert committee consolidates all the previous versions of the scale and the final Spanish version of the SSS-W (SSS-W-E) was agreed by consensus. Experts had a minim of 20 years of clinical experience and were associate university professor, and researchers. Consensus was reached by the nominal group technique in 2 sessions (2 h by session). Finally, cognitive interviews were completed in a convenience sample of 15 women who confirmed the readability and comprehensibility of the items. This procedure aimed to obtain the instrument’s face validity. No changes were implemented following the interviews with the women.

### 2.2. Description of the SSS-W-E

The final version of the SSS-W-E developed by the authors comprises 30 items measured on a five-point Likert scale with response options ranging from 1 = strongly disagree to 5 = strongly agree. The SSS-W-E is divided into 5 dimensions or domains comprising 6 items each, namely contentment, communication, compatibility, concern about the relationship and personal concerns. The score range for each domain is 6–30, and it is calculated by adding the scores of the individual items comprising each separate dimension. The SSS-W-E global score is calculated by summing up the scores of the 5 domains (Contentment + Communication + Compatibility + (Relational Concern + Personal Concern/2)) obtaining ranges from 24 to 120.

### 2.3. Sampling and Study Population

This study was carried out in a family planning clinic in Logroño (La Rioja, Spain) using a cross-sectional design.

Minimum sample size was estimated at 300 following Vet et al. [26] criteria that recommends a minimum of 10 subjects per item. Sexually active women aged ≥ 16, who attended the family planning clinic from June 2020 to February 2021, were recruited consecutively to participate in this study. We excluded women who did not speak Spanish, those who could not complete the scale due to mental or other disorders and those who did not give their consent to participate in this study. In total, 316 women signed the consent form and were enrolled in the study. The data were collected by three midwives who were trained for this purpose.

### 2.4. Data Collection

All the participants completed the SSS-W-E. In addition, a sociodemographic questionnaire was designed ad hoc to describe the characteristics of the sample. This tool included the following sociodemographic, reproductive and sexual variables: age, nationality, number of children, level of education, income, employment situation, affective-sexual relationship, stability of the relationship, cohabiting with the partner and frequency of sexual activity.

### 2.5. Data Analysis

Sociodemographic and clinical variables were analyzed using descriptive statistics, that is, mean and standard deviation (SD) for quantitative variables and frequency for categorical ones. In addition, descriptive statistics, including mean, SD, skewness and kurtosis were used to describe the participants’ responses and summarize the global score of the scale.

Psychometric analysis of the SSS-W-E included reliability and validity tests. We analyzed internal consistency by calculating Cronbach’s alpha coefficient of the total scale and each dimension separately, accepting values of 0.70 or higher as an indicator of good internal consistency [27,28]. The Kaiser–Meyer–Olkin (KMO) test was used to determine the sampling adequacy of data; the sampling adequacy for the analysis was confirmed if KMO > 0.6. The Bartlett’s sphericity test was used to compare the correlation matrix to the identity matrix, accepting a significance value < 0.05.

Exploratory factor analysis (EFA) was performed using principal component analysis with a Varimax rotation to determine the number of latent constructs and the underlying factorial structure of the SSS-W-E’s domains. Two complementary criteria were used in order to estimate the number of factors on the scale: (1) the Kaiser–Guttman or latent root criterion, (2) the drop contrast criterion [29,30].

We performed all statistical analyses using SPSS Software version 23 (IBM Corporation, New Orchard Road Armonk, New York, NY, USA).

### 2.6. Ethical Considerations

The information was treated confidentially and anonymously since they had dissociated data, following the Data Protection Regulation (EU) 2016/679 of the European Parliament and the Spanish Organic Law 3/2018. The researchers do not declare any type of ethical, moral, or legal conflict, nor do they claim to have received financial compensation of any other kind. The participants did not receive any type of compensation for answering the questionnaire, as it was voluntary. The study was approved by the ethics committee of the Rioja Biomedical Research Center (CIBIR) [31] (reference CEImLar P.I. 386).

## 3. Results

The sociodemographic, reproductive and sexual characteristics of the sample are shown in Table 1. The scale was completed by 316 women aged 17–50 (mean age 33.4 and SD ± 8.6). Almost half of our sample (48.4%) did not have any children. The majority of the women were Spanish (82.9%) and more than 60% were trained to vocational or university level (68.7%). In total, 56% earned between EUR 12,000 and 35,000 annually, 56.6% worked for others and 33.0% were either unemployed or studying. Only 4.7% had sexual intercourse every day, while just over half of the participants said that they had sexual intercourse monthly or occasionally.

Mean, SD, skewness and kurtosis values for the SSS-W-E are presented in Table 2. Most of the items followed a normal distribution, without excessive skewness and kurtosis. The items with the highest scores were item 7 “My partner often gets defensive when I try discussing sex” and item 27 “I’m worried that my sexual difficulties might cause me to seek sexual fulfillment outside my relationship”. The lowest scores were recorded for item 12 “My partner has no difficulty talking about their deepest feelings and emotions when I want him to” and item 20 “I am worried that my sexual difficulties will adversely affect my relationship”.

Internal consistency of the global SSS-W-E and each separate dimension was excellent. Cronbach’s alpha for the total scale was 0.93; Cronbach’s alpha values for each dimension were 0.86 contentment, 0.70 communication, 0.81 compatibility, 0.90 relational concern and 0.93 personal concern.

The results from KMO and Bartlett’s sphericity tests suggested that factor analysis was suitable for this test. KMO values for the whole scale and each dimension separately were 0.92, and ranged from 0.76 to 0.88, respectively. Bartlett’s sphericity test was significant (*p* < 0.01) for the global scale and each dimension separately.

**Figure 1:**
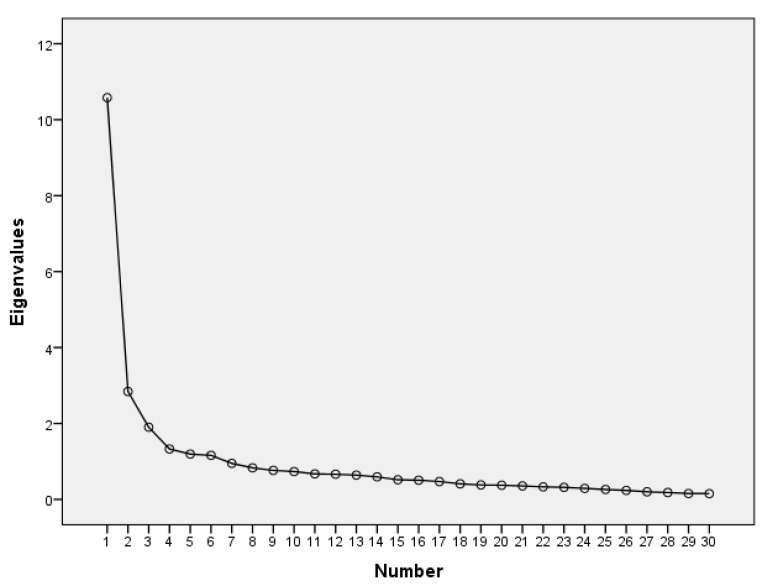
Sedimentation graph of the SSS-W-E.

The Kaiser–Guttman or latent root criterion identified five factors with eigenvalues greater than 1 as shown in Table 3, which explained 60.05% of the total variance of the items. The second criterion, fall contrast or screen test, also showed the presence of five factors through the sedimentation graph, as shown in Figure 1.

EFA was performed using principal component analysis with a Varimax rotation, considering the following criteria: factor load > 0.30, number of items per factor according to the original [16], the Traditional Chinese [17], and the Portuguese [19] versions, the interpretability of the results and the theory that supports the SSS-W. According to these criteria, it is observed that the five conceptual domains of the original SSS-W adapt well to SSS-W-E in its Spanish version (Table 4). The personal domain “contentment” included items 1–6; the relational domains (communication and compatibility) integrated items 7–18; the domain “concern about the relationship” grouped items 19–24; the domain “personal concerns” comprised items 25–30. In the matrix of rotated components (Varimax) presented in Table 4, it is observed that the elements load significantly in the five previous factors. As in the previous three versions of the SSS-W, the relational domains “communication” and “compatibility” comprised items 7–18. However, in the SSS-W-E, the “communication” dimension related better to items 7–13 and 15, whereas the “compatibility” dimension included items 8,14,16,17 and 18.

## 4. Discussion

In this study we present the results from the transcultural adaptation and validation of the SSS-W-E. The SSS-W-E is culturally equivalent to the original instrument and will allow Spanish clinicians and researchers to evaluate Spanish women’s personal and relational sexual satisfaction through five domains: contentment, communication, compatibility, relational concern, and personal concern. To our knowledge, this is the first study to adapt and validate the original SSS-W for use in the Spanish population. Similar validation studies have been conducted in Taiwanese women [17], Taiwanese women with breast cancer [18], and Brazilian women [19].

No language difficulties were found during the cross-cultural adaptation process; however, some expressions were slightly modified to guarantee cultural equivalence of the Spanish version of the tool. None of the 316 women who took part in the validation study had any difficulty understanding and completing the SSS-W-E.

Regarding the characteristics of the participants, in our study, the range and average age of the Spanish women, 33 years, is very similar to that of the American and Brazilian women. However, it is much lower than the age of Taiwanese women, 48 years old, of whom more than a third were between 50 and 60 years old. In terms of educational level, 60% of the Spanish women had a university education, 62–67% of the American women, 80% of the Brazilian women and only 22.3% of the Taiwanese women. Furthermore, we know that 33.6% of the Spanish women and 60% of the Brazilian women do not live with their partner and that most of the American women were not married, while 96.1% of the Taiwanese women were. In addition, the Spanish and Brazilian study sample does not integrate two distinct groups of women with and without sexual dysfunction as in the American and Taiwanese studies. The characteristics of the women interviewed in the validation studies of the different versions may have determined some of the particularities found in the construct validity of the scale. In our study, the number of dimensions coincides with the original American version, but there are differences in the number of items in the communication and compatibility dimensions.

Internal consistency of the global SSS-W-E, and of each of its five domains, was high [28], with values getting close to those found in the original, Traditional Chinese and Portuguese versions. The dimension that showed the lowest internal consistency was communication, with a Cronbach’s alpha value of 0.70. Similarly, in the Portuguese and American version the communication domain is the one showing the lowest internal consistency 0.70 and 0.74, respectively. We agree with Meston et al. (2005) in their appreciation that the internal consistency value of 0.70 for the communication domain is satisfactory considering that the items that compose the domain are very few for a very broad content [16].

In our study, the five domains explained 60.05% of the total variance of the scale, close to the 63% found in the original US version, but lower than the Traditional Chinese version where the five factors explained 77.5% of the variance.

Like the original and Portuguese versions of the SSS-W, EFA of the SSS-W-E in its Spanish version identified five domains. Unlike these three versions of the tool, the SSS-W in its Traditional Chinese version comprises only four domains, after the domains personal concern and concern about the relationship were merged. This difference could be due to cultural factors. Specifically, while the amorous and erotic imaginary of European and American women share cultural, literary, and film-loving influences, which have probably contributed to a closer conceptualization of sexual satisfaction [32], the cultural influences of Taiwanese women may have a different root. While in European and American culture falling in love and romantic love, which fills the one who experiences it with joy, are highly valued socially at the beginning of an affective relationship, in Taiwanese culture affective relationships may have other more valued functions such as strengthening family or economic alliances, leaving more personal interests in second place. This may at least partially explain why Taiwanese women’s sexual well-being seems to depend on the sexual well-being of their partners to a greater extent than that of American and European women. The latter seem to be more concerned with their own sexual well-being [17].

Principal component analysis using the Varimax rotation method showed some particular characteristics. Specifically, the distribution of items 7–18 was different from that observed in the original and Portuguese versions of the SSS-W. Whereas, in the original and Portuguese versions these items were evenly distributed between the “communication” and “compatibility” domains, in the Spanish version the “communication” domain comprised seven items (7,9–13, and 15) and the “compatibility” domain comprised five items (8,14,16,17 and 18). In particular, item 8 “My partner and I do not discuss sex openly enough with each other or do not discuss sex often enough” was integrated into the “compatibility” domain, whilst items 13 and 15 “I often feel my partner isn’t sensitive or aware enough about my sexual likes and desires” and “I often feel that my partner’s beliefs and attitudes about sex are too different from mine” were integrated into the “communication domain”. Both the “communication” and “compatibility” domains make up the relational aspects of sexual satisfaction. It is possible that Spanish women do not conceive that one can occur without the other. In recent years, the Spanish population has undergone very significant social [33] and legislative [34] changes, aimed at promoting a more equitable relationship between men and women. Although more traditional sexual models, guided by Catholic morality, still coexist with other more heterogeneous ones, more and more Spanish women demand their right to sexuality without a power relationship, and value the possibility of negotiating discrepancies in terms of sexual frequency, practices and permissible sexual games [33,35,36]. Sexual compatibility is built through good verbal and non-verbal communication, where both partners share tastes, beliefs, values and attitudes towards sex [37].

Being able to openly express one’s sexual desires and address sexual concerns with one’s partner can be seen as advantageous, especially when the partner has different preferences and expectations [38]. Accordingly, Spanish women seem to understand that compatibility with their partner increases when they can talk openly about sex [39,40], and that communication improves when their partner knows and respects their sexual tastes and desires, and agreements about sexual beliefs and attitudes are reached. The slight differences found between the SSS-W-E and the SSS-W in its original and Portuguese versions do not substantially change the use of the scale, but emphasize the need for not only linguistic but also cultural adaptation and validation of measurement tools, especially when they involve concepts as complex and culturally dependent as sexuality.

## 5. Limitations and Strengths

The sample used for data validation is large enough to guarantee an adequate representation of Spanish women in the reproductive age group (aged 17–50). However, younger and older women were not represented in this sample. Therefore, we recommend that the SSS-W-E is validated in Spanish women under 17 and over 50 to extend the use of this scale to Spanish women of any age. Another characteristic of our sample is that we did not exclude any woman because of her sexual orientation or gender identity or because she maintains an affective-sexual relationship different from the traditional ones where the couple lives together and their relationship is stable, to obtain a better representation of Spanish women. However, as a result of sampling among women seeking counseling at a family planning center, it is possible that there was an overrepresentation of cisgender and heterosexual women in the sample introducing a selection bias. Although our aim was to prove that this scale is suitable for use in Spanish women by measuring its consistency and construct validity, the assessment of other measures such as convergent or divergent validity would have increased the study’s quality.

## 6. Conclusions

The results from this study of transcultural adaptation and validation of the SSS-W indicate that the tool in its Spanish version has good overall reliability and validity. Our findings are largely compatible with the initial hypothesis, which make the SSS-W-E a useful tool for the evaluation of women’s sexual satisfaction in clinical practice and research, in Spain. However, the ability of SSS-W-E to discriminate between women with and without sexual dysfunction has not been evaluated in this study. Caution is needed regarding the generalization of the use of this instrument. Future studies will have to validate its suitability for use in groups of Spanish women with specific characteristics of age, illness, disabilities, sexual orientation or gender identity.

## Figures and Tables

**Table 1:** Sociodemographic characteristics (*n* = 316).

Variables	N	%
Age		
− 17–20 years old	16	5.10%
− 21–30 years old	121	38.30%
− 31–40 years old	108	34.10%
− 41–50 years old	71	22.50%
Number of Children		
− None	153	48.40%
− One	57	18.00%
− Two	81	25.60%
− Three or more	25	8.00%
Nationality		
− Spanish	262	82.90%
− Latin American	34	10.00%
− European	13	4.10%
− Others	7	2.20%
Level of Education		
− None	1	0.30%
− Primary School (up to 12 years old)	17	5.40%
− Middle School (up to 16 years)	31	9.80%
− High School (up to 18 years old)	50	15.80%
− Vocational training	34	10.80%
− University degree	183	57.90%
Annual Income (Euro)		
− < EUR 12.000	73	23.10%
− From EUR 12.001 to 20.000	98	31%
− From EUR 20.001 to 35.000	79	25%
− From EUR 35.001 to 60.000	48	15.20%
− From EUR 60.001 to 100.000	16	5.10%
− More than EUR 100.000	2	0.60%
Employment situation		
− Unemployed	90	28.50%
− Employed	179	56.60%
− Self-Employed	33	10.40%
− Student	14	4.50%
Stability of the relationship		
− Stable relationship	268	84.80%
− Unstable relationship	48	15.20%
Living with your partner		
− Yes	210	66.40%
− No	106	33.60%
Frequency of Sexual Activity		
− Occasional (once or several times a year)	74	23.40%
− Monthly (once or several times a month)	97	30.70%
− Weekly (once or several times a week)	130	41.20%
− Daily (once or several times a day)	15	4.70%

**Table 2:** Descriptive statistics of the items of SSS-W-E.

Item	Mean	SD	Asymmetry	Kurtosis
Item 1	3.65	±1.150	−0.643	−0.329
Item 2	3.32	±1.350	−0.269	−1.146
Item 3	3.78	±1.286	−0.644	−0.911
Item 4	3.35	±1.273	−0.301	−0.979
Item 5	3.33	±1.429	−0.345	−1.233
Item 6	3.64	±1.212	−0.651	−0.548
Item 7	4.29	±1.059	−1.469	1.351
Item 8	3.56	±1.539	−0.557	−1.244
Item 9	4.10	±1.066	−1.036	0.233
Item 10	4.01	±1.108	−0.978	0.136
Item 11	3.97	±1.189	−0.884	−0.384
Item 12	3.27	±1.326	−0.142	−1.170
Item 13	3.79	±1.247	−0.724	−0.582
Item 14	3.80	±1.356	−0.845	−0.530
Item 15	4.06	±1.158	−1.057	0.146
Item 16	3.83	±1.262	−0.806	−0.426
Item 17	3.90	±1.397	−0.892	−0.664
Item 18	3.97	±1.202	−1.011	0.035
Item 19	3.34	±1.333	−0.142	−1.241
Item 20	3.30	±1.398	−0.131	−1.381
Item 21	3.77	±1.343	−0.729	−0.714
Item 22	3.31	±1.354	−0.183	−1.266
Item 23	3.80	±1.325	−0.713	−0.783
Item 24	3.41	±1.369	−0.261	−1.197
Item 25	3.54	±1.347	−0.454	−1.051
Item 26	3.57	±1.300	−0.469	−0.923
Item 27	4.22	±1.129	−1.330	0.661
Item 28	3.66	±1.346	−0.567	−0.944
Item 29	3.49	±1.364	−0.392	−1.155
Item 30	3.73	±1.314	−0.682	−0.755

**Table 3:** Total variance explained by the five dimensions of the Spanish version of the Sexual Satisfaction Scale for Women (SSS-W-E). Rotation sum of charges squared.

Component	Total	% Variance	% Accumulate
1	4.156	13.855	13.855
2	4.149	13.832	27.686
3	3.611	12.037	39.723
4	3.595	11.982	51.705
5	2.203	8.343	60.049

**Table 4:** Rotated component matrix. Principal component analysis (Varimax).

Item	Factor 1	Factor 2	Factor 3	Factor 4	Factor 5
Item 1	0.771				
Item 2	0.640				
Item 3	0.319				
Item 4	0.733				
Item 5	0.591				
Item 6	0.793				
Item 7		0.545			
Item 8			0.600		
Item 9		0.496			
Item 10		0.691			
Item 11		0.456			
Item 12		0.527			
Item 13		0.622			
Item 14			0.695		
Item 15		0.632			
Item 16			0.329		
Item 17			0.630		
Item 18			0.515		
Item 19				0.680	
Item 20				0.625	
Item 21				0.480	
Item 22				0.820	
Item 23				0.644	
Item 24				0.749	
Item 25					0.719
Item 26					0.751
Item 27					0.432
Item 28					0.773
Item 29					0.800
Item 30					0.810

## Data Availability

Data are available contacting with corresponding author.
